# Effect of HELLP syndrome on acute kidney injury in pregnancy and pregnancy outcomes: a systematic review and meta-analysis

**DOI:** 10.1186/s12884-020-03346-4

**Published:** 2020-10-30

**Authors:** Qiang Liu, Guan-jun Ling, Shao-quan Zhang, Wen-qing Zhai, Yi-juan Chen

**Affiliations:** Maternity and Child Health Care & Red Cross Hospital of Qinzhou, Qinzhou, 535099 Guangxi China

**Keywords:** HELLP syndrome, Acute kidney injury, Pregnancy outcomes, AKI

## Abstract

**Background:**

HELLP syndrome may increase adverse pregnancy outcomes, though the incidence of it is not high. At present, the impact of HELLP syndrome on P-AKI (acute kidney injury during pregnancy) and maternal and infant outcomes is controversial. Thus, we conducted a meta-analysis to find out more about the relationship between HELLP syndrome and P-AKI and pregnancy outcomes.

**Methods:**

We systematically searched PubMed, Embassy and Cochrane Databases for cohort studies and RCT to assess the effect of HELLP syndrome on P-AKI and maternal and infant outcomes. Study-specific risk estimates were combined by using fixed-effect or random-effect models.

**Results:**

This meta-analysis included 11 cohort studies with a total of 6333 Participants, including 355 cases of pregnant women with HELLP syndrome and 5979 cases that without. HELLP syndrome was associated with relatively higher risk of P-AKI (OR4.87 95% CI 3.31 ~ 7.17, P<0.001), fetal mortality (OR1.56 95% CI 1.45 ~ 2.11, P<0.001) and Maternal death (OR3.70 95% CI 1.72 ~ 7.99, P<0.001).

**Conclusions:**

HELLP syndrome is associated with relatively higher risk of P-AKI, fetal mortality and maternal death.

## Background

At present, high blood pressure caused by pregnancy is still an important public health problem in the world. Up to 500,000 pregnant women die every year due to complications related to preeclampsia [1]. HELLP syndrome was defined by Weistein in 1982 as a clinical manifestation of elevated liver enzymes, hemolysis, and thrombocytopenia [2]. For pregnant women, HELLP syndrome is a serious pregnancy complication that can threaten the safety of the mother and fetus. According to reports in the literature, the incidence of HELLP syndrome in patients with pregnancy-induced hypertension range from 2 to 19.3%, while the prenatal mortality rate can reach from 7 to 60% [3]. Studies have reported that among patients with preeclampsia, patients with HELLP syndrome have shorter gestational age, lower fetal weight, and higher fetal mortality than those without HELLP syndrome [4, 5].

In patients with HELLP syndrome, acute kidney injury is a common cause of maternal and fetal death. For pregnant women,acute kidney injury can lead to anuria, heart failure, and severe pulmonary edema [6]. These complications can bring about rapid death of pregnant women and fetuses. At present, the relationship between HELLP syndrome and acute kidney injury in pregnancy is controversial. Many studies believe that acute kidney injury during pregnancy and pregnancy outcomes are related to HLEPP syndrome [7]. However, there are also studies that have different opinions, and they believe that acute kidney injury in pregnancy is related to preeclampsia rather than HELLP syndrome [8]. Therefore, a systemic analysis of the relationship between HELLP syndrome and P-AKI and pregnancy outcomes is necessary.

We collected studies on patients with hypertension during pregnancy, and performed a meta-analysis on those with or without HELLP syndrome. We used meta-analysis to assess the association between HELLP syndrome and acute kidney injury to help clinicians make better decisions. To our knowledge, this is the first meta-analysis of HELLP syndrome and Acute kidney injury during pregnancy (P-AKI). Our research has been registered with PROSPERO, the code is CRD42018112333.

## Methods

Based on PRISMA standards and procedures, we used the following method for meta-analysis [9]. We searched PubMed, Embassy, The Cochrane database for related cohort studies and RCT. The retrieval time was from the establishment of the database till May 2019. Our search used a combination of controlled vocabulary and natural language terms, and the references included in the related literatures were also been reviewed. Search terms included: HELLP syndrome/pregnant women/AKI or P-AKI/Neonatal outcomes/Fetal outcomes/stillbirth/perinatal outcomes/Eclampsia/Preeclampsia. Cross-sectional, descriptive or case series/reports were excluded. Studies on pregnant women with chronic kidney disease or lithangiuria were also excluded.

### Data extraction and quality assessment

The content mainly includes: 1. Basic information included in the research: the author’s name, year of publication, country.2. The basic characteristics of the study subjects: Definition of acute kidney injury, Definition of HELLP syndrome, sample size, Pregnancy outcomes and quantity score.3. The main data of clinical outcomes, and confounding factors which need to be adjusted. Two researchers (LQ and LGJ) screened the literature independently, extracted the data and cross-checked. In case of a disagreement, we would resolved by discussion or submitted it to the third researcher (ZSQ) to make a final decision. We assessed the authenticity and quality of the included studies using Newcastle-Ottawa scales (NOS) [10], and 6 points or more was defined as high quality research.

### Statistical analysis

Meta-analysis was performed with State software version 12.0. The effect index of dichotomous data was the risk ratio (OR), and the effect index of the continuous data was the mean difference (MD), and there was also a 95% confidence interval (95% CI) [11]. We used an χ 2 test (test level α = 0.1) to assess the heterogeneity of the included studies, and the quantitative analysis was done combined with I2 [11–13]. If there is no statistical heterogeneity across the studies, a fixed effect model was used for meta-analysis, but if there is statistical heterogeneity, the source of heterogeneity would be further analyzed. When the obvious clinical heterogeneity was excluded, a random effects model was used for the meta-analysis. If there was significant clinical heterogeneity, a subgroup analysis or sensitivity analysis, or only a descriptive analysis was used. The level of the meta-analysis was set to α = 0.05.

## Results

**Fig. 1:**
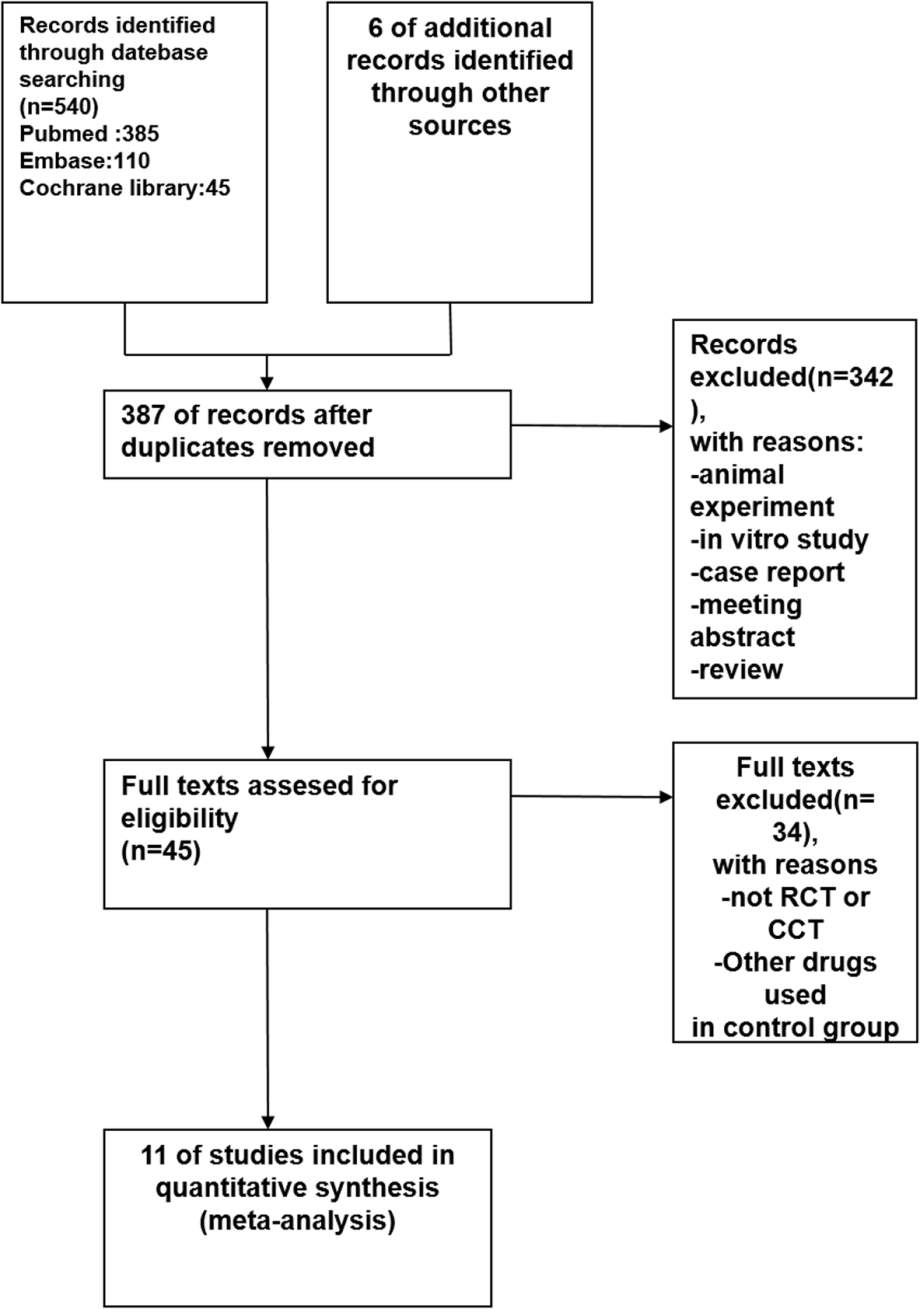
Process for identifying studies eligible for the meta-analysis

The literature search yielded 546 articles, with 11 studies identified according to the inclusion criteria (Fig. 1) [4, 7, 8, 14–21]. The characteristics of these included studies were summarized in Tables 1 and 2. These studies were performed from 1993 to 2011 with sample sizes ranging from 60 to 1099. The primary disease in all the studies was gestational hypertension. The patients were divided into HELLP syndrome group and no HELLP syndrome group and the study was conducted between the two groups. The study-design types were as follows: retrospective studies 9 items [7, 8, 14, 15, 17–21], and prospective studies 2 items [4, 16].

**Table 1:** Characteristics of the included studies

Author, Year	Study Design	Country	HELLP was defined	AKI (ARF) was defined	Sample size of patients with AKI in patients with HELLP^**a**^	Sample size of patients with AKI in patients without HELLP ^**a**^	Major clinical outcomes	Quality score
**Gul 2004** [7]	**RC**	**Turkey**	**The criteria of Sibai**	**creatinine level ≥ 1.2 mg/dL and/or oliguria < 400 mL/24 h**	132 (20/112)	156 (10/146)	**Kidney outcome、**	**6**
**Zuberi 1998** [14]	**RC**	**Pakistan**	**The criteria of Sibai**	Not clear	38 (4/34)	38 (1/37)	**Kidney outcome、Fetal outcome、**	**5**
**Yildirim 2011** [15]	**RC**	**Turkey**	Not clear	**a creatinine clearance ≤ 20 mL/min was present with an elevated serum creatinine level ≥ 2 mg/dL**	196 21/175	903 14/889	**Kidney outcome、Fetal outcome**	**6**
**F. Abroug 1992** [16]	**PC**	**Tunisia**	**hemolysis, increased liver enzymes and thrombocytopenia.**	**diagnosed on an increased creatinine level over 160 umol/1**	12 (8/4)	50 (15/35)	**Kidney outcome, Fetal outcome**	**6**
**Haddad 2000** [5]	**RC**	**USA**	**The criteria of Sibai**	**oligouria or anuria in association with creatinine clearance ≤ 20 mL/min and an elevated serum creatinine level ≥ 2 mg/dL**	30 (1/29)	30 (0/30)	**Kidney outcome**	**6**
**Liu 2006** [17]	**RC**	**China taiwan**	**Hemolysis, elevated liver enzymes, low platelet count**	**severe reduction in renal function with elevated serum** **creatinine greater than 120** μmol**/L** (**> 1.4 mg/dL**)**.**	52 (14/38)	212 (7/205)	**Kidney outcome**	**7**
**Martin 1993** [18]	**RC**	**USA**	**the presence of thrombocytopenia, hepatic dysfunction, and haemolysis**	Not clear	62 (2/60)	55 (0/55)	**Kidney outcome**	**6**
**Turgut 2010** [19]	**RC**	**Turkey**	**the presence of thrombocytopenia, hepatic dysfunction, and haemolysis**	**creatinine clearance of ≤ 20 mL/min and an elevated serum creatinine level of ≥ 2 mg/dL**	111 (9/102)	467 (11/456)	**Kidney outcome**	**6**

The criteria of Sibai: hemolysis, elevated lactate dehydrogenase (LDH > 600 IU/L), aspartate (AST > 40 IU/L), and/or alanine aminotransferase (ALT > 40 IU/L) and low platelet (Plt) count as class III (Plt:100–150 × 10 3 /mL), class II (Plt:50–99 × 10 3 /mL), and class I (Plt < 50 × 10 3 /mL)

*Abbreviations*: *CC* indicates case–control, *RC* retrospective cohort, *PC* prospective cohort, *CV* cardiovascular

*AKI* Acute kidney injury,

^a^ Expressed as total number of patients (number in HELLP group/number in control group)

**Table 2:** Characteristics of the included studies

Author,Year	Study Design	Country	HELLP was defined	Sample size of patients with stillbirth in patients with HELLP^**a**^	sample size of patients with stillbirth in patients without HELLP^**a**^	Major clinical outcomes	Quality score
**Gul 2005** [4]	**PC**	**Turkey**	**The criteria of Sibai**	106 (11/95)	261 (12/249)	**Fetal outcome**	**6**
**Abramovici 1999** [20]	**RC**	**Pakistan**	**The criteria of Sibai**	133 (10/123)	141 (5/136)	**Fetal outcome**	**6**
**Osmanağaoğlu 2004** [21]	**RC**	**Turkey**	**Hemolysis, elevated liver enzymes, low platelet count**	51 27/24	52 19/23	**Fetal outcome**	**5**

^a^ Expressed as total number of patients (number in PR-AKI group/number in control group)

### Kidney outcomes

**Fig. 2:**
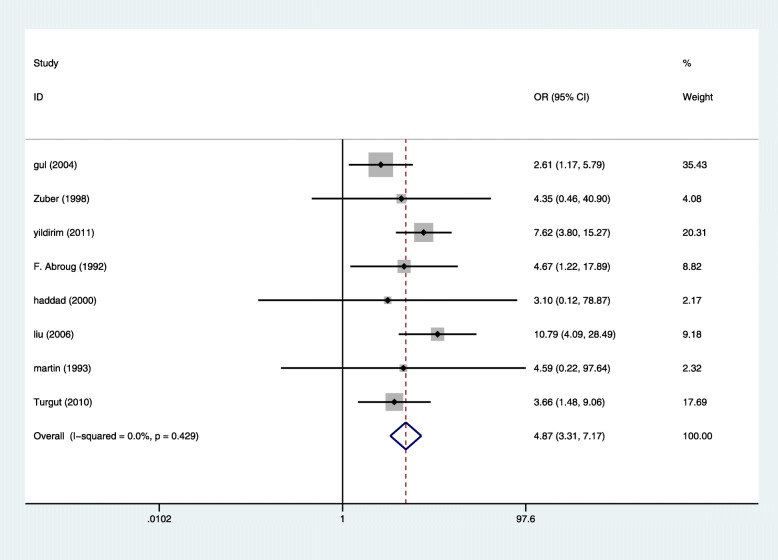
Comparison of the incidence of AKI in pregnant women with HELLP syndrome and no HELLP syndrome

Eight studies reported 79 cases of AKI in 556 Pregnant women with HELLP syndrome and 58 cases of AKI in 1158 pregnant women without HELLP syndrome, producing a 4.87 fold (95% CI 3.31 to 7.17, *P* = 0.000) higher likelihood in pregnant women with HELLP syndrome [7, 8, 14–19], with Very low evidence of heterogeneity (I2 = 0%, *P* = 0.429 Fig. 2).

### Pregnancy outcomes

**Fig. 3:**
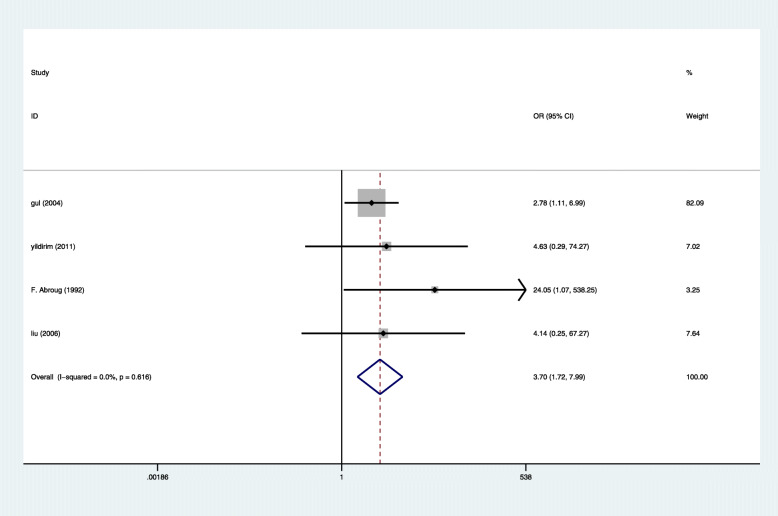
Comparison of the Maternal death in pregnant women with HELLP syndrome and no HELLP syndrome

**Fig. 4:**
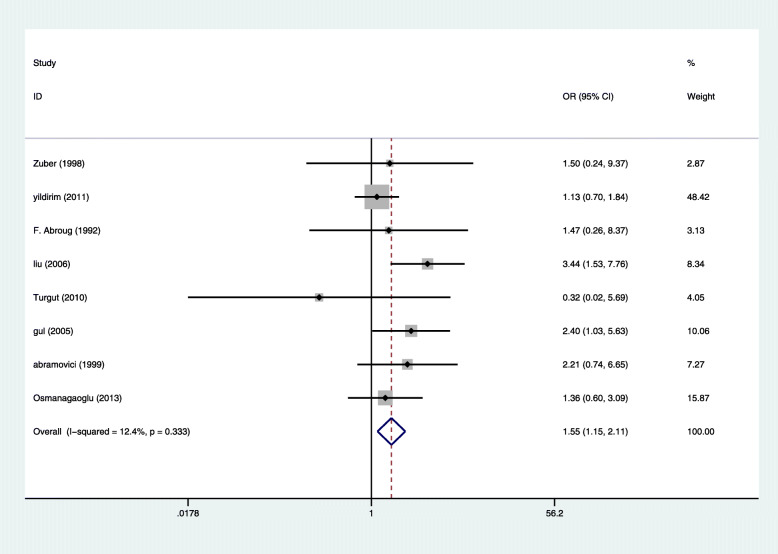
Comparison of the stillbirth in pregnant women with HELLP syndrome and no HELLP syndrome

**Fig. 5:**
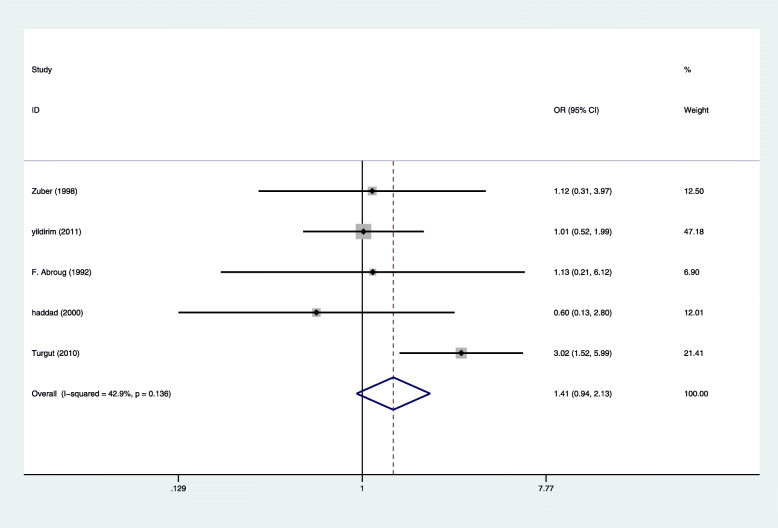
Comparison of Neonatal death in pregnant women with HELLP syndrome and no HELLP syndrome

Four studies reported 11 cases of maternal death in 280 pregnant women with HELLP syndrome and 32 cases of maternal death in 1149 pregnant women without HELLP syndrome, producing a 3.70 fold (95% CI 1.72 to 7.99, *P* = 0.001) higher likelihood in pregnant women with HELLP syndrome [7, 15–17], with Very low evidence of heterogeneity (I2 = 0%, *P* = 0.616 Fig. 3). Eight studies reported 87 cases of stillbirth in 612 pregnant women with HELLP syndrome and 163 cases of stillbirth in 1997 pregnant women without HELLP syndrome [4, 14–17, 19–21], producing a 1.56 fold (95% CI 1.45 to 2.11, *P* = 0.005) higher likelihood in pregnant women with HELLP syndrome, with Very low evidence of heterogeneity (I2 = 12.4%, *P* = 0.333 Fig. 4). There was not enough evidence that pregnant women with HELLP syndrome are associated with an increased incidence of neonatal death (OR, 1.41; 95% CI 0.94 to 2.13; *P* = 0.098(Fig. 5). When a single study was removed in sequence, the heterogeneity did not decrease significantly and the conclusion did not change.

### Publication bias

**Fig. 6:**
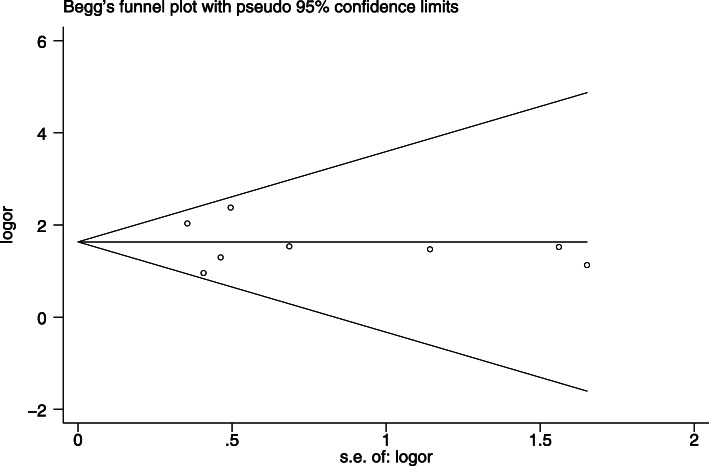
Begg’s test about Fig. 2

The Newcastle-Ottawa scales (NOS) evaluation indicated that the incidence rate of AKI had low-quality evidence (Table 1). There may be biases in the studies included,but the symmetry of the funnel plot was further evaluated using Begg’s test,and no publication bias was found (Begg’s test, *P* = 0.38) (Fig. 6).

## Discussion

HELLP syndrome is considered to be an important risk factor that increases the mortality of pregnant women and fetuses [16]. The occurrence of acute kidney injury during pregnancy makes the original condition aggravated and complicated [22]. We searched for publications on the association of HELLP syndrome with AKI and pregnancy outcomes. These studies have different opinions on the relationship between HELLP syndrome and AKI. We hope to unify this understanding through meta-analysis. We used heterogeneity to assess the size of the difference between each study, and thus decided whether to use a random effects model or a fixed effects model. In our meta-analysis, the heterogeneity between studies was not significant (I2 < 50%), so we did not perform subgroup analysis and meta-regression analysis. We used Begg’s test and funnel plot to assess the bias of the study, but no publication bias was found. All the above show that our research results are stable and reliable, which can help clinicians make decisions.

To the best of our knowledge, this is the first meta-analysis to explore the effect of HELLP syndrome on P-AKI and pregnancy outcomes. It suggests that pregnant women with HELLP syndrome was associated with higher risk of AKI (4.87 fold), stillbirth (3.70 fold), and maternal death (1.56 fold). The effect of HELLP syndrome on neonatal mortality was not statistically significant in this study, but our data on this subject was less, an in-depth study with more data is necessary. In our study, the stillbirth rate of pregnant women with HELLP syndrome was 49.5%, which was higher than that reported in Serdar study (7.4–34%) [23]. Premature birth and placental abruption are the main causes of stillbirth. Moreover, the maternal mortality rate in our study was 2.5% higher than that in Sibai (1%) literature [24].

Traditionally, HELLP syndrome is considered to be a variant of preeclampsia, but it is, in fact, a specific disease, since 20% of pregnant women with HELLP syndrome do not have a history of hypertension or proteinuria [25, 26]. The etiology of HELLP syndrome is not fully understood. Immunological changes, platelet aggregation, endothelial dysfunction, arterial hypertension, and an inborn error of fatty acid oxidative metabolism have all been suggested as potential etiological factors [27]. Studies have shown that levels of anti-angiogenic factors (sFlt-1 and sEng) elevated and concentrations of pro-angiogenic mediators (PIGF) decreased in pregnant women with HELLP syndrome [28]. HELLP syndrome seems to be a TMA-like disorder, they have similar clinical manifestations such as mechanical hemolysis, thrombocytopenia, and AKI [29]. A recent study suggests that there may be a link between HELLP syndrome and complement dysregulation [30]. The cause of acute kidney injury caused by HELLP syndrome is not clear. There are studies reported that in patients with HELLP syndrome with acute kidney injury, most of the kidney pathological biopsy is acute tubular necrosis (ATN), and a small part is acute renal cortical necrosis (ARCN). The pathological results of acute tubular necrosis (ATN) and acute renal cortical necrosis (ARCN) both show microvascular injury and microthrombosis [31–33]. HELLP syndrome causes intravascular platelet activation and microvascular endothelial damage, which are concerned by scholars, because these are the basis of intravascular microthrombosis [32]. Some scholars also believe that the subsequent development of endothelial injury and thrombotic microangiopathies in patients with HELLP syndrome leading to renal tubular necrosis is a pathogenic process that may cause acute renal failure [33]. The kidney injury rate of patients with HELLP syndrome is about 10%, and about 10–40% of acute kidney injury patients require hospitalization for dialysis [34, 35].

### Clinical implications and limitations

The strength of this systematic review and meta-analysis lies in the instruction significance for clinical questions, as a large volume of data was included and a rigorous methodology used. However, there are some limitations to our study. The first is that the sample size of some included studies is small, which would lead to bias; Second, since individual studies have different definitions of AKI and HELLP syndrome, some patients with AKI and HELLP syndrome may be missed. It is currently believed that the diagnosis criteria of HELLP syndrome proposed by Sibai et al. is stricter and more popular [36]. In the assessment of acute kidney injury, AKIN and RIFLE are widely used [37].

## Conclusion

HELLP syndrome is associated with relatively higher risk of P-AKI, fetal mortality and maternal death. Although some conclusions require more research to support, this study resolves the dispute.

## Data Availability

All data can be obtained from the manuscript.

## Abbreviations

AKI: acute kidney injury
P-AKI: acute kidney injury during pregnancy
HELLP: hemolysis, elevated liver enzymes and low platelet (count)
RCT: randomized clinical trial
NOS: Newcastle-Ottawa scales
OR: risk ratio
MD: mean difference
TMA: thrombotic microangiopathy
ATN: acute tubular necrosis
ARCN: acute renal cortical necrosis

## References

[1] Gedik E, Yucel N, Sahin T, Koca E, Colak YZ, Togal T (2017). Hemolysis, elevated liver enzymes, and low platelet syndrome: outcomes for patients admitted to intensive care at a tertiary referral hospital. Hypertens Pregnancy.

[2] Weinstein L (1982). Syndrome of hemolysis, elevated liver enzymes, and low platelet count: a severe consequence of hypertension in pregnancy. Am J Obstet Gynecol.

[3] Celik C, Gezginc K, Altintepe L, Tonbul HZ, Yaman ST, Akyurek C, Turk S (2003). Results of the pregnancies with HELLP syndrome. Ren Fail.

[4] Gul A, Cebeci A, Aslan H, Polat I, Ozdemir A, Ceylan Y (2005). Perinatal outcomes in severe preeclampsia-eclampsia with and without HELLP syndrome. Gynecol Obstet Investig.

[5] Haddad B, Barton JR, Livingston JC, Chahine R, Sibai BM (2000). Risk factors for adverse maternal outcomes among women with HELLP (hemolysis, elevated liver enzymes, and low platelet count) syndrome. Am J Obstet Gynecol.

[6] Cavkaytar S, Ugurlu EN, Karaer A, Tapisiz OL, Danisman N (2007). Are clinical symptoms more predictive than laboratory parameters for adverse maternal outcome in HELLP syndrome?. Acta Obstet Gynecol Scand.

[7] Gul A, Aslan H, Cebeci A, Polat I, Ulusoy S, Ceylan Y (2004). Maternal and fetal outcomes in HELLP syndrome complicated with acute renal failure. Ren Fail.

[8] Haddad B, Barton JR, Livingston JC, Chahine R, Sibai BM (2000). HELLP (hemolysis, elevated liver enzymes, and low platelet count) syndrome versus severe preeclampsia: onset at < or =28.0 weeks' gestation. Am J Obstet Gynecol.

[9] Liberati A, Altman DG, Tetzlaff J, Mulrow C, Gøtzsche PC, Ioannidis JP, Clarke M, Devereaux PJ, Kleijnen J, Moher D (2009). The PRISMA statement for reporting systematic reviews and meta-analyses of studies that evaluate health care interventions: explanation and elaboration. PLoS Med.

[10] Wells G, Shea B, O'connell D, Peterson J, Welch V, Losos M and Tugwell P. The Newcastle-Ottawa Scale (NOS) for assessing the quality of nonrandomised studies in meta-analyses. Ottawa (ON): Ottawa Hospital Research Institute; 2009.

[11] Higgins JP, Thompson SG, Deeks JJ, Altman DG (2003). Measuring inconsistency in meta-analyses. Bmj.

[12] DerSimonian R, Laird N (1986). Meta-analysis in clinical trials. Control Clin Trials.

[13] Begg CB, Mazumdar M. Operating characteristics of a rank correlation test for publication bias. Biometrics. 1994:1088–101. 7786990

[14] Zuberi NF, Arif K, Khan FM, Pal JA (1998). A comparison of severe pre-eclampsia/eclampsia in patients with and without HELLP syndrome. J Pakistan Med Assoc.

[15] Yıldırım G, Güngördük K, Aslan H, Gül A, Bayraktar M, Ceylan Y (2011). Comparison of perinatal and maternal outcomes of severe preeclampsia, eclampsia, and HELLP syndrome. J Turkish German Gynecol Assoc.

[16] Abroug F, Boujdaria R, Nouira S, Abroug S, Souissi M, Najjar M, Secourgeon J, Bouchoucha S (1992). HELLP syndrome: incidence and maternal-fetal outcome—a prospective study. Intensive Care Med.

[17] Liu CM, Chang SD, Cheng PJ, Chao AS (2006). Comparisons of maternal and perinatal outcomes in Taiwanese women with complete and partial HELLP syndrome and women with severe pre-eclampsia without HELLP. J Obstet Gynaecol Res.

[18] Martin JN, Perry KG, Miles JF, Blake PG, Magann EF, Roberts WE, Martin RW (1993). The interrelationship of eclampsia, HELLP syndrome, and prematurity: cofactors for significant maternal and perinatal risk. BJOG Int J Obstet Gynaecol.

[19] Turgut A, Demirci O, Demirci E, Uludoğan M (2010). Comparison of maternal and neonatal outcomes in women with HELLP syndrome and women with severe preeclampsia without HELLP syndrome. J Prenatal Med.

[20] Abramovici D, Friedman SA, Mercer BM, Audibert F, Kao L, Sibai BM (1999). Neonatal outcome in severe preeclampsia at 24 to 36 weeks' gestation: does the HELLP (hemolysis, elevated liver enzymes, and low platelet count) syndrome matter?. Am J Obstet Gynecol.

[21] Osmanağaoğlu MA, Erdoğan İ, Zengin Ü, Bozkaya H (2004). Comparison between HELLP syndrome, chronic hypertension, and superimposed preeclampsia on chronic hypertension without HELLP syndrome. J Perinat Med.

[22] Drakeley AJ, Le Roux PA, Anthony J, Penny J (2002). Acute renal failure complicating severe preeclampsia requiring admission to an obstetric intensive care unit. Am J Obstet Gynecol.

[23] Aydin S, Ersan F, Ark C, Arıoğlu Aydın Ç (2014). Partial HELLP syndrome: maternal, perinatal, subsequent pregnancy and long-term maternal outcomes. J Obstet Gynaecol Res.

[24] Sibai BM, Ramadan MK (1993). Acute renal failure in pregnancies complicated by hemolysis, elevated liver enzymes, and low platelets. Am J Obstet Gynecol.

[25] Sibai BM (2004). Diagnosis, controversies, and management of the syndrome of hemolysis, elevated liver enzymes, and low platelet count. Obstet Gynecol.

[26] Barton JR, Sibai BM. Gastrointestinal complications of pre-eclampsia. Semin Perinatol. 2009;33(3):179–88. 10.1053/j.semperi.2009.02.006. 10.1053/j.semperi.2009.02.006 19464509

[27] Yildirim G, Gungorduk K, Gul A, Asicioglu O, Sudolmus S, Gungorduk OC, Ceylan Y (2012). HELLP syndrome: 8 years of experience from a tertiary referral center in western Turkey. Hypertens Pregnancy.

[28] Joshi D, James A, Quaglia A, Westbrook RH, Heneghan MA (2010). Liver disease in pregnancy. Lancet.

[29] Kuklina EV, Ayala C, Callaghan WM (2009). Hypertensive disorders and severe obstetric morbidity in the United States. Obstet Gynecol.

[30] Fakhouri F, Jablonski M, Lepercq J, Blouin J, Benachi A, Hourmant M, Pirson Y, Dürrbach A, Grünfeld J-P, Knebelmann B (2008). Factor H, membrane cofactor protein, and factor I mutations in patients with hemolysis, elevated liver enzymes, and low platelet count syndrome. Blood.

[31] Sibai BM, Taslimi MM, el-Nazer A, Amon E, Mabie BC, Ryan GM: Maternal-perinatal outcome associated with the syndrome of hemolysis, elevated liver enzymes, and low platelets in severe preeclampsia-eclampsia. Am J Obstet Gynecol 1986, 155(3):501–509. 10.1016/0002-9378(86)90266-8 3529964

[32] Ghosh AK, Vashisht K, Varma S, Khullar D, Sakhuja V (1994). Acute renal failure in a patient with HELLP syndrome--an unusual complication of eclampsia. Ren Fail.

[33] Abraham KA, Kennelly M, Dorman AM, Walshe JJ (2003). Pathogenesis of acute renal failure associated with the HELLP syndrome: a case report and review of the literature. Eur J Obstet Gynecol Reprod Biol.

[34] Fang JT, Chen YC, Huang CC (2000). Unusual presentation of mesangial proliferative glomerulonephritis in HELLP syndrome associated with acute renal failure. Ren Fail.

[35] Gupta A, Ferguson J, Rahman M, Weber-Shrikant E, Venuto R (2012). Acute oliguric renal failure in HELLP syndrome: case report and review of literature. Ren Fail.

[36] Sibai BM (1990). The HELLP syndrome (hemolysis, elevated liver enzymes, and low platelets): much ado about nothing. Am J Obstet Gynecol.

[37] Liu Y, Ma X, Zheng J, Liu X, Yan T (2017). Pregnancy outcomes in patients with acute kidney injury during pregnancy: a systematic review and meta-analysis. BMC Pregnancy Childbirth.

